# The aldosteronoma resolution score predicts the prognosis of hypertension under two surgical procedures

**DOI:** 10.3389/fendo.2025.1651381

**Published:** 2025-09-19

**Authors:** Yifan Huang, Qianhao Huang, Yijie Xie, Chaoyue Ouyang, Yuedong Chen, Chao Huang

**Affiliations:** ^1^ The School of Clinical Medicine, Fujian Medical University, Fuzhou, China; ^2^ The Key Laboratory of Urinary Tract Tumors and Calculi, Department of Urology Surgery, The First Affiliated Hospital of Xiamen University, School of Medicine, Xiamen University, Xiamen, China

**Keywords:** primary aldosteronism, Aldosteronoma Resolution Score (ARS), hypertension resolution, partial adrenalectomy, total adrenalectomy

## Abstract

**Background:**

Primary aldosteronism (PA) is a leading cause of secondary hypertension, and aldosterone-producing adenoma (APA) is the most prevalent subtype. Surgical intervention is the primary treatment modality. However, a subset of patients fails to achieve normotension postoperatively. The Aldosteronoma Resolution Score (ARS), a preoperative predictive model, was used to forecast postoperative hypertension resolution. This study aims to validate the accuracy of the ARS in predicting hypertension resolution in Asian patients undergoing different surgical approaches.

**Methods:**

We conducted a retrospective review of 129 patients diagnosed with APA who underwent adrenal surgery. After excluding patients with incomplete data or insufficient follow-up, 83 patients were included in the analysis. Patients were stratified into two groups based on surgical approach: partial adrenalectomy and total adrenalectomy. Clinical and blood pressure data were collected at baseline and 6 months postoperatively.

**Results:**

At the 6-month follow-up, 62.65% (52/83) of patients achieved normotension without medication, while 37.35% (31/83) required continued antihypertensive therapy. Multivariate logistic regression analysis identified that patients achieving complete hypertension resolution were significantly more likely to have: preoperative use of ≤2 antihypertensive medications, hypertension duration ≤6 years, and BMI ≤25 kg/m². The predictive accuracy of the ARS, evaluated using the area under the receiver operating characteristic curve (AUC), was 0.866 for the overall cohort. Among the 49 patients (59.0%) who underwent partial adrenalectomy, 36.73% required antihypertensive medication, with an ARS AUC of 0.839. Among the 34 patients (41.0%) who underwent total adrenalectomy, 61.76% achieved normotension, with an ARS AUC of 0.897. No significant difference was observed in postoperative blood pressure outcomes between the two surgical approaches (*P* = 0.889), nor did the surgical approach affect the predictive accuracy of the ARS (*P* = 0.461).

**Conclusion:**

Our study validates the ARS, originally derived from Western populations, as a reliable preoperative predictor of hypertension resolution following adrenalectomy in Asian patients with APA. Furthermore, both partial and total adrenalectomy demonstrated comparable efficacy in achieving blood pressure control. These findings support the broad applicability of the ARS across diverse patient populations and surgical approaches.

## 1 Introduction

Primary aldosteronism (PA) was first reported by the American endocrinologist Conn in 1955 (1). PA is typically caused by aldosterone - producing adenomas (APA) or bilateral adrenal hyperplasia (IHA) (2, 3). As a significant etiology of secondary hypertension, the actual prevalence of PA is underestimated by at least 3–5 times (4). Its harm stems from the excessive activation of the mineralocorticoid receptor, which leads to target - organ damage. Typical clinical manifestations include hypervolemia, hypernatremia, and hypokalemia. PA increases the risks of cardiovascular and renal diseases, as well as mortality (5). Thus, the prognosis of PA is a major concern for clinicians.

Regarding clinical prediction models for hypertension resolution after adrenalectomy, several options exist—including the PASO score, Utsumi nomogram, and GRAAS score. Among these, the Aldosteronoma Resolution Score (ARS) is distinguished by its simplicity and minimal data requirements. While the PASO criteria offer comprehensive multidimensional outcomes, the ARS was selected for this validation study due to its practical utility in preoperative decision-making, leveraging readily available routine clinical parameters (6).

The Aldosterone Resolution Score (ARS) was proposed by Zarnegar (7) et al. This scoring system comprehensively takes into account the patient’s body mass index (BMI), gender, duration of hypertension, and the number of preoperative antihypertensive medications. It has demonstrated good external validation performance. Further external validation studies by Gustavo Romero - Velez (8) et al. confirmed that the ARS score has reliable clinical predictive accuracy in Hispanic and Black patient populations. Huang - Fu (9) et al. suggested that the ARS scoring system holds certain value in predicting the postoperative hypertensive status of patients with primary aldosteronism. These findings highlight the ARS score as a convenient and reliable clinical tool, enabling physicians to predict the likelihood of complete resolution of hypertension following adrenalectomy based on routine clinical data.

For patients with aldosterone-producing adenoma (APA) and unilateral adrenal hyperplasia (UAH), surgical treatment is the main intervention. Currently, the choice of surgical procedures focuses on weighing the advantages and disadvantages between partial adrenalectomy and total adrenalectomy. Since the team of Janetschek (10) first applied laparoscopic adrenal - preserving surgery to the treatment of APA in 1997, partial adrenalectomy has received much attention because it can maximize the preservation of functional adrenal tissue. Its advantage lies in maintaining the adrenal hormone reserve and retaining the ability to regulate physiological stress. However, this surgical procedure has the risk of tumor residue or recurrence and requires extremely high precision from the surgical operator. Incomplete hemostasis during the operation may increase the risk of postoperative bleeding complications. In contrast, although total adrenalectomy can completely remove the lesion, it may lead to adrenal insufficiency and result in a deficiency of glucocorticoids and mineralocorticoids. This, in turn, can cause long - term complications such as osteoporosis, abnormal glucose metabolism, Addisonian crisis, and androgen deficiency in women. It can seriously affect the quality of life of patients.

The core of the current controversy regarding the choice of surgical procedures for APA lies in how to strike the best balance between radical cure of the disease and preservation of adrenal function. This study aims to construct a framework for the choice of surgical procedures based on evidence and provide a theoretical basis for clinical precise decision by comparing the differences between the two surgical procedures in terms of the level of postoperative blood pressure control.

## 2 Methods

### 2.1 Patient selection

**Figure 1:**
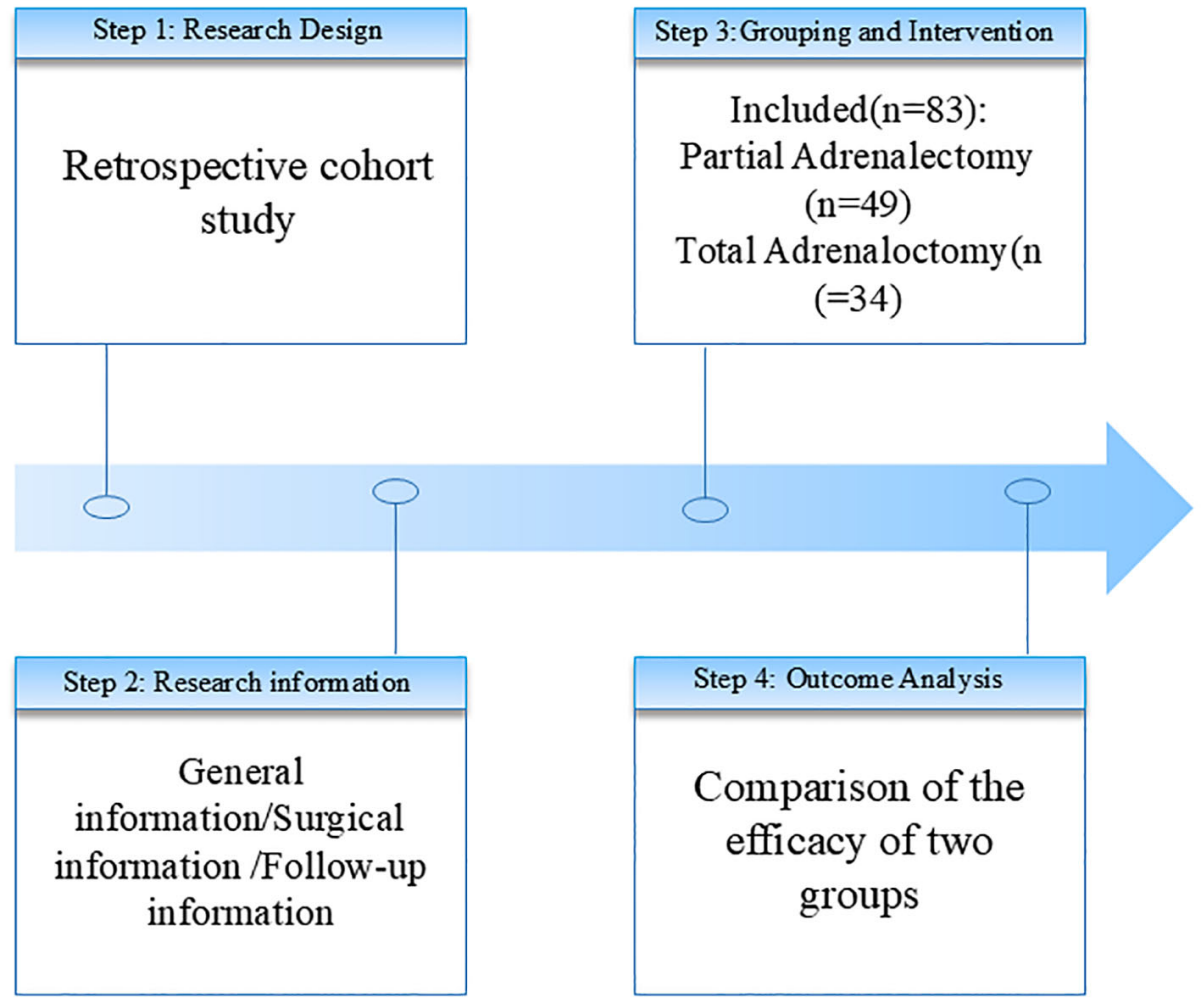
Study flow chart.

We conducted a retrospective investigation on clinical variables associated with complete hypertension resolution in patients treated at the First Affiliated Hospital of Xiamen University. Initially, 129 patients diagnosed with aldosterone-producing adenoma (APA) who underwent surgical treatment (total or partial adrenalectomy) were considered. After excluding 46 patients due to missing/incomplete data or follow-up <6 months, 83 patients were included in the final analysis. Diagnostic criteria for APA were as follows: (1) History of antihypertensive medication resistance (± hypokalemia), accompanied by elevated plasma aldosterone concentration and suppressed plasma renin activity (PRA); (2) Biochemical confirmation via aldosterone-to-renin ratio (ARR) ≥30 ng/dL/ng/mL/h, with additional validation through high-salt diet loading, fludrocortisone suppression, saline infusion, or captopril challenge tests; (3) Differentiation from bilateral hyperplasia using computed tomography (CT), magnetic resonance imaging (MRI), and adrenal venous sampling (AVS). We performed AVS sequentially under unstimulated conditions, with selectivity confirmed by a selectivity index (SI) >2.0 and lateralization defined by a lateralization index (LI) >4.0 based on aldosterone/cortisol ratios (6). Exclusion criteria included: (1) Coexisting secondary hypertension;(2) Pregnancy, severe infection/stress, hepatic/renal dysfunction, autoimmune diseases, or malignancy;(3) Non-compliant follow-up (**Figure 1**).

### 2.2 Definitions

We classified blood pressure at baseline according to the 1999 World Health Organization guidelines, with normal defined as <140/90 mmHg. For postoperative assessment, office BP was measured three times after 5 minutes of seated rest using validated oscillometric devices (Omron HEM-7322), with the average recorded. Patients were considered to have achieved hypertension resolution if they maintained BP <140/90 mmHg without antihypertensive medications at 6 months postoperatively. Patients were classified as having persistent hypertension if they met one of the following two conditions within 6 months after surgery: (1) Systolic BP ≥140 mmHg or diastolic BP ≥90 mmHg; (2) Continued requirement for antihypertensive therapy. The 6-month time point was chosen as the endpoint for review because the effects of adrenalectomy and mineralocorticoid receptor antagonists on blood pressure diminish or become minimal within this period (7, 11).

### 2.3 Data collection

Data such as age, sex, BMI, family history of hypertension in first - degree relatives, smoking history, duration of preoperative hypertension, highest blood pressure, number of preoperative antihypertensive medications, serum potassium levels, and maximum diameter of clinical tumors were collected from medical records.

### 2.4 Prediction model

The Aldosterone Resolution Score (ARS) was used as the prediction model. This scoring system incorporates the patient’s BMI, sex, duration of hypertension, and the number of preoperative antihypertensive medications. Patients were assigned 1 point each for BMI ≤25 kg/m², female sex, or hypertension duration ≤6 years, and 2 points for taking ≤2 types of antihypertensive medications preoperatively. The ARS ranges from 0 to 5 points. A higher ARS score indicates a greater likelihood of hypertension resolution after adrenalectomy. An ARS score ≥4 predicts a high probability of complete hypertension cure.

### 2.5 Statistical analysis

Results are reported as medians or means ± standard deviation (SD). We performed univariate analysis to estimate the correlation between complete hypertension resolution and preoperative variables, including sex, BMI, age, family history, smoking history, duration of hypertension, highest blood pressure, number of preoperative antihypertensive medications, serum potassium levels, and tumor volume. We compared continuous parametric variables by using the t - test, non - parametric variables by using the Mann - Whitney U test, and categorical variables by using the chi - square test. If the expected cell counts were <5, we would use fisher’s exact test instead.

After selecting candidate variables based on univariate analysis, we conducted multivariate logistic regression analysis to identify clinical predictors associated with complete hypertension resolution.

Subsequently, to validate the ARS, we adopted the cutoffs proposed by Zarnegar et al. to dichotomize four continuous variables—age ≤55 years, BMI ≤25 kg/m², duration of hypertension ≤6 years, and number of antihypertensive medications ≤2. We further conducted collinearity testing to exclude interdependent variables and keep the model independent. Additionally, we used the area under the curve (AUC) derived from the receiver operating characteristic (ROC) curve to evaluate the predictive accuracy of the ARS in our institutional dataset. All statistical analyses were performed using SPSS 27.

## 3 Results

At the 6 - month follow - up, 62.65% of patients achieved normal blood pressure without the need for antihypertensive medications, while 37.35% of patients still required medication for blood pressure control, indicating either reduced blood pressure or a decreased need for antihypertensive drugs post - adrenalectomy. Preoperative hypokalemia was observed in 73 patients. The 83 patients had a mean age of 46 ± 10 years, 35% were male, 37% had a family history of hypertension, and 17% had a smoking history. The mean BMI was 24 ± 3 kg/m². The median duration of preoperative hypertension was 4 years (range: 0–30 years), and the median number of preoperative antihypertensive medications was 2 (range: 1–5). The maximum tumor diameter was 2 cm. Post - adrenalectomy, 52 out of 83 patients (63%) achieved complete hypertension resolution, while the remaining 37% showed improved blood pressure control but still required antihypertensive medications (median: 0, range: 0–2) (**Table 1**).

**Table 1:** Univariate analyses of candidate predictor variables in the dataset of The First Affiliated Hospital of Xiamen University.

Variable	Resolved (N = 52)/%	Not resolved (N = 31)/%	P value
Age, y	40.5	53	<0.01
Sex, female, n (%)	35 (67.3)	10 (32.3)	0.14
BMI, kg/m²	22.9 ± 2.4	25.2 ± 3.3	0.03
Family history of hypertension, n (%)	14 (26.9)	11 (35.5)	0.111
History of smoking, n (%)	5 (9.6)	7 (22.6)	0.023
Duration of hypertension, y (range)	1.5 (0-30)	8 (0-20)	<0.01
No. of preoperative antihypertensive medications, n (range)	2 (1-4)	3 (1-5)	<0.01
Systolic blood pressure, mm Hg	171.6(17)	179.0(22.0)	0.176
Diastolic blood pressure, mm Hg	105.8(12)	113.5(24.9)	0.525
Maximum diameter of tumor, cm	2	2	0.38
Tumor location, left side, n (%)	30 (57.7)	19 (61.3)	0.749
Blood potassium, mmol/L (range)	2.55 (1.5-5.0)	2.8 (2.0-4.6)	0.013

summarizes the independent preoperative predictors associated with complete hypertension resolution in the univariate analysis. The following variables were significantly correlated with hypertension outcomes: age, BMI, smoking history, duration of hypertension, number of preoperative antihypertensive medications, and serum potassium levels (P < 0.05) (**Table 1**).

Multivariate logistic regression analysis revealed that in this cohort. The number of preoperative antihypertensive medications ≤2 (OR = 6.86, 95% CI = 2.37–19.86, P <0.01), duration of hypertension ≤6 years (OR = 13.44, 95% CI = 4.56–39.64, P < 0.01), and BMI ≤25 kg/m² (OR = 3.94, 95% CI = 1.47–10.55, P = 0.034) could serve as predictive factors for hypertension resolution. However, female sex (OR = 3.20, 95% CI = 1.25–8.22, P = 0.414) was not a significant predictor of resolution. In this analysis, the following three variables were identified as independent predictors of complete hypertension resolution: preoperative use of ≤2 antihypertensive medications, duration of hypertension ≤6 years, and BMI ≤25 kg/m² (**Table 2**).

**Table 2:** Results of multivariate logistic regression analysis of the 4 major predictor variables.

Variable	Odds ratio	95%CI	P value
BMI ≤25 kg/m2	3.94	1.47-10.55	0.034
≤2 antihypertensive medications	6.86	2.37-19.86	<0.01
≤6 years of hypertension	13.44	4.56 -39.64	<0.01
Sex,female	3.20	1.25-8.22	0.414
Family history of hypertension	5.11	1.01-25.91	0.049
Age ≤ 55 years	4.91	1.36-17.68	0.486
Blood potassium ≤ 3.5 mmol/L	3.27	0.96-11.12	0.165
History of smoking	3.85	1.15-12.83	0.388

**Figure 2:**
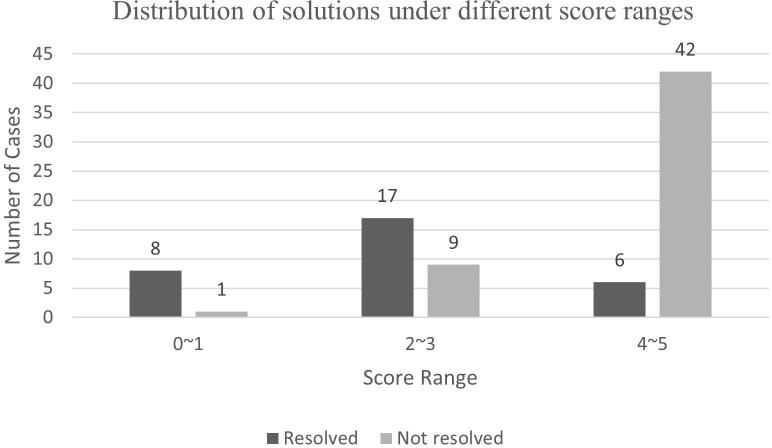
The application performance of the aldosteronoma resolution score in patients with primary aldosteronism adenoma at The First Affiliated Hospital of Xiamen University. The proportions of patients with complete and incomplete resolution are correlated with this predictive score.

**Figure 3:**
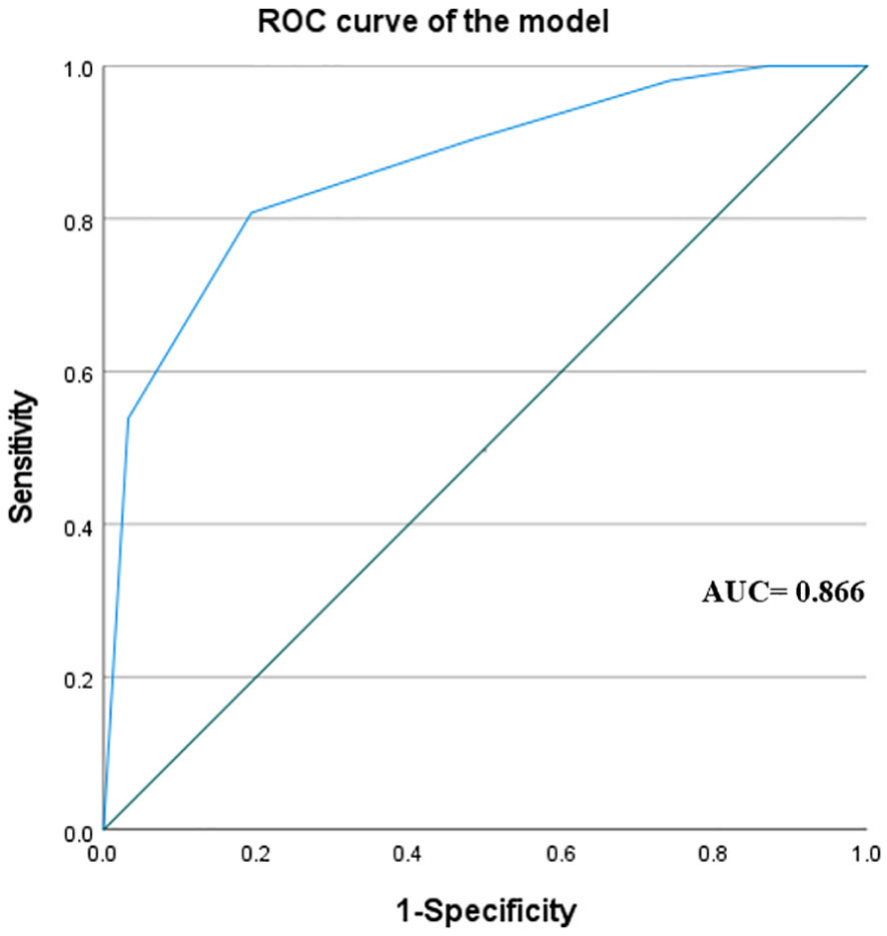
The receiver operator characteristics (ROC) curve of the aldosteronoma resolution score in The First Affiliated Hospital of Xiamen University. The value of the area under the curve is 0.866.

After using the ARS for scoring, we found that among patients with an ARS of 0–1, 1 out of 9 patients (11%) achieved complete hypertension resolution. Among patients with an ARS of 2–3, 9 out of 26 patients (35%) achieved complete hypertension resolution. Among patients with an ARS of 4–5, 42 out of 48 patients (88%) achieved complete hypertension resolution (**Figure 2**). To determine the accuracy of the model, the ROC curve analysis revealed an area under the curve (AUC) of 0.866 (**Figure 3**).

In the context of surgical treatment, it is crucial to understand the impact of different surgical approaches on blood pressure outcomes. Therefore, we focused on the dynamic recovery of blood pressure following two mainstream surgical approaches. Clinical and blood pressure data were assessed at baseline and during the 6 - month follow - up. We found that in the total adrenalectomy group, 21 patients (61.76%) achieved normal blood pressure, whereas in the partial adrenalectomy group, 18 patients (36.73%) still required antihypertensive medications for blood pressure control. Analysis of the data revealed that the difference in postoperative blood pressure outcomes between the two surgical methods was not significant (P = 0.889) (**Table 3**).

**Table 3:** The corresponding relationship between surgical resection methods and blood pressure status.

	Blood pressure status		Total
	Resolved	Not resolved	
Total Excision	21	13	34
Partial Excision	31	18	49
Total	52	31	83

**Figure 4:**
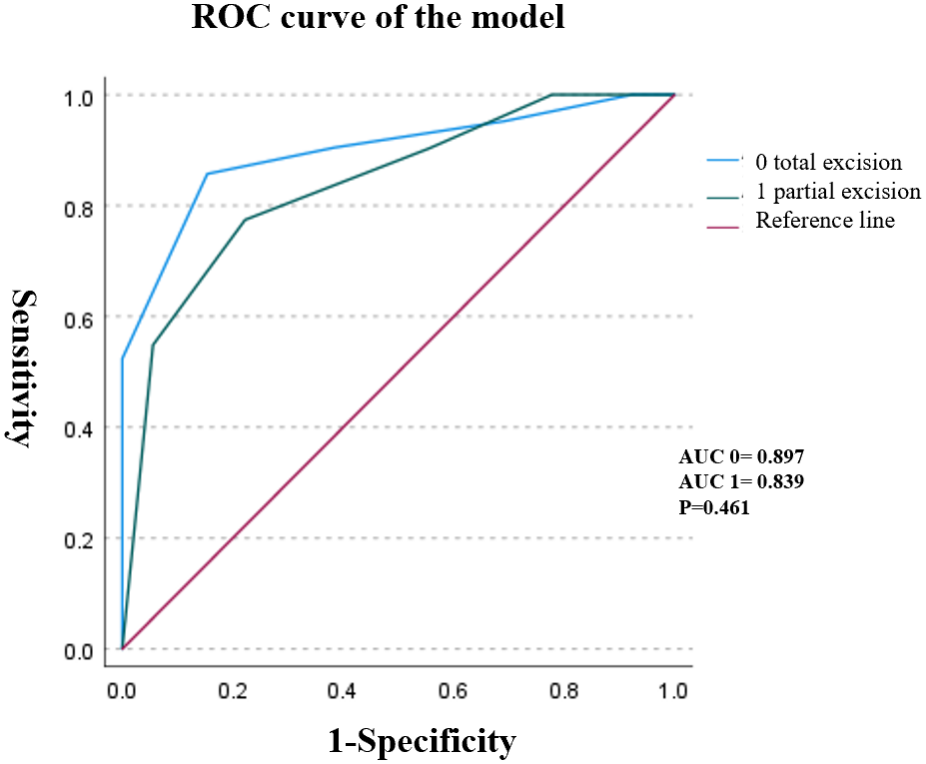
The receiver - operator characteristic (ROC) curves under different surgical methods showed that there were no statistically significant differences in patient prognosis among different surgical methods (P = 0.461).

Subsequently, we explored the accuracy of the ARS scoring system across different surgical approaches. Through data analysis, we found that the ARS scoring system’s ability to reflect patient prognosis did not show significant differences between the surgical methods (P = 0.461). This further underscores the advantage of the ARS scoring system in demonstrating excellent generalizability (**Figure 4**).

## 4 Discussion

Primary aldosteronism (PA), particularly aldosterone - producing adenoma (APA), is a common cause of secondary hypertension and poses a significant burden on the heart and kidneys. Silvia Monticone (12) et al., through meta - analysis, demonstrated that compared to patients with essential hypertension, those with PA have a significantly higher risk of stroke, coronary artery disease, atrial fibrillation, and heart failure. Additionally, PA markedly increases the risk of diabetes, metabolic syndrome, and left ventricular hypertrophy. Beyond cardiac fibrosis and heart failure, PA also contributes to progressive renal damage through mechanisms such as renal fibrosis, vascular disease, and podocyte injury (13–15). Currently, laparoscopic adrenalectomy is widely regarded as the preferred treatment for unilateral adrenal disease according to the Endocrine Society Clinical Practice Guidelines (16) and the Japanese Endocrine Society Guidelines (17). It has well-established therapeutic efficacy in managing hypertension and its complications (18).

In 2008, Zarnegar (7) et al. developed a predictive model to estimate the probability of complete hypertension resolution post - surgery based on patient sex, BMI, duration of hypertension, and the number of preoperative antihypertensive medications. We could use the Aldosterone Resolution Score (ARS) derived from this model to stratify patients into low - score (ARS 0–1), intermediate - score (ARS 2–3), and high - score (ARS 4–5) groups, with predictive accuracies of 27%, 46%, and 75%, respectively.

In our study, univariate analysis identified preoperative BMI, smoking history, duration of hypertension, number of preoperative antihypertensive medications, and preoperative serum potassium levels as independent predictors of complete hypertension resolution. The relationship between smoking and hypertension is particularly complex. Although a direct causal link between smoking and PA has not been established, extensive research indicates that smoking exacerbates hypertension and its target - organ damage through multiple mechanisms (6, 19).

Multivariate analysis revealed that the use of ≤2 preoperative antihypertensive medications, a duration of hypertension ≤6 years, and a BMI ≤25 kg/m² were independently associated with complete hypertension resolution. Previous retrospective studies have frequently mentioned these variables. Although Zarnegar (7) et al. noted that sex was significantly associated with hypertension resolution, it did not emerge as an independent predictor in our multivariate analysis. This discrepancy may be attributed to the relatively small sample size of our study or the possibility that the effect of sex on hypertension resolution was confounded or moderated by other variables, which may have been overshadowed by more significant factors in our analysis. This discrepancy may be attributed to the relatively small sample size of our study, or the possibility that the effect of sex on hypertension resolution was moderated by other variables. This may have been overshadowed by more significant factors in our analysis.

Zarnegar (7) et al. externally validated the ARS using cross - tabulation, reporting a negative predictive value (NPV) of 72.4% for patients with ARS scores of 0 or 1 and a positive predictive value (PPV) of 75.0% for those with ARS scores of 4 or 5. Our results were consistent, with an AUC of 0.866 confirming the high predictive accuracy of the ARS. Further analysis revealed no significant difference in postoperative blood pressure outcomes between total and partial adrenalectomy (P = 0.889). Multiple studies have reported that both surgical approaches are safe and feasible during the perioperative period. Melih Balci (20) et al. compared the clinical outcomes of partial and total adrenalectomy. They concluded that partial adrenalectomy is safe and effective, with similar therapeutic outcomes to total adrenalectomy, while also eliminating the need for lifelong steroid replacement therapy. Sheng - Fu Chen (21) et al. supported this view by comparing perioperative and postoperative parameters between the two procedures. In recent years, partial adrenalectomy has gained popularity due to its tissue-preserving advantages. While unilateral total adrenalectomy rarely causes adrenal insufficiency in patients with normal contralateral glands, partial resection eliminates even this minimal risk and prevents the theoretical need for glucocorticoid replacement during major physiological stressors (22). More importantly, it preserves adrenal androgen production in women and maintains future adrenal reserve in cases of contralateral pathology (23). Kun - Peng Li (24) et al., through a meta - analysis of 834 patients, demonstrated no significant differences in postoperative serum aldosterone, hypokalemia, potassium levels, ARR, or renin levels between the two surgical approaches.

Furthermore, our validation of the ARS scoring system across different surgical approaches demonstrated its robust generalizability. The ARS system provides a stable and effective tool for assessing patient prognosis, offering valuable guidance for surgical selection and outcome prediction. This tool enables doctors to more comprehensively and accurately evaluate patient progression and recovery expectations during treatment.

Despite the progress made in this research, several limitations remain. The relatively low incidence of primary aldosteronism (PA) led to a limited sample size during the external validation of the Adrenal Response Score (ARS) using retrospective data. It potentially affects the accuracy of the research results. Moreover, direct comparison with the validation study by Zarnegar (7) et al. is challenging as they did not use the area under the curve (AUC) value as the validation indicator for ARS.

In clinical practice, many PA patients who have undergone adrenalectomy are concerned about whether their blood pressure can return to normal and whether they can discontinue antihypertensive drugs. The ARS meets this clinical need. Notably, although the ARS scoring system originated from Western research, our external validation study indicates its effectiveness in accurately identifying Asian patients and predicting postoperative hypertension remission. Based on routine medical history and physical examination data, the ARS can predict postoperative hypertension remission, making it a user - friendly and practical tool for clinical decision - making.

In future research, we plan to expand the sample size and extend the follow-up period to further verify the predictive value of ARS for hypertension prognosis. This will provide more accurate and comprehensive research results, offering stronger support for clinical practice and patient management.

## 5 Conclusion

Our findings indicate that the ARS, originally developed using Western data, is a reliable tool for predicting postoperative blood pressure outcomes in Asian patients with APA. Both partial and total adrenalectomy demonstrated comparable efficacy in hypertension resolution (P = 0.889), and the surgical approach did not influence the predictive accuracy of the ARS (P = 0.461). These results support the applicability of the ARS in diverse patient populations and surgical contexts.

## Data Availability

The datasets used and/or analyzed during the current study are available from the corresponding author upon reasonable request.
